# New Insight into a Classic Stem Cell: the Satellite Cell may Communicate with the Muscle Fiber via Extracellular Vesicles—A Perspective on “Fusion-Independent Satellite Cell Communication to Muscle Fibers During Load-Induced Hypertrophy”

**DOI:** 10.1093/function/zqaa015

**Published:** 2020-09-03

**Authors:** Zipora Yablonka-Reuveni, Christoph Lepper

**Affiliations:** 1 Department of Biological Structure, School of Medicine, University of Washington, 1959 NE Pacific Street, Box 357420, Seattle, WA 98195, USA; 2 Department of Physiology & Cell Biology, College of Medicine, The Ohio State University, 1645 Neil Ave., Columbus, OH 43210, USA

Plasticity of skeletal muscle allows the tissue to rapidly remodel in response to changing environmental conditions to meet the new physiologic demand. This remodeling can include alterations in muscle fiber type distribution and/or changes in muscle strength, with or without muscle hypertrophy. Resistance training, ie, mechanical overload, especially, can lead to dramatic increases in muscle mass. The molecular regulation of hypertrophy is highly complex involving several mechanisms including endocrine and paracrine signaling, mechanotransduction, and satellite cells as well as nonmyogenic cells.^1^ The role of satellite cells in the context of muscle overload-induced hypertrophy is of particular interest. Satellite cells derive their name from their unique localization, ie, residing on the surface of the myofiber between the myofiber plasma membrane and its surrounding basal lamina. Transplantation, lineage-tracing, and cell ablation studies have helped cement the satellite cells’ essential role as muscle stem cells, which readily provide new myonuclei for regeneration of multinucleated myofibers after acute injury.^2^ Evidence on the contribution of satellite cells to load-induced hypertrophy only recently emerged. While an initial report indicated that muscle mass gains appear to be possible without the accretion of new myonuclei via fusion of satellite cells,^3^ a subsequent study showed that fusion-competent satellite cells are necessary for robust gains in muscle mass.^4^

In the inaugural issue of *FUNCTION*, Murach et al. report that satellite cells release extracellular vesicles (EVs) that are taken up by the myofiber during load-induced hypertrophy and conclude that this process contributes to modulating muscle mass.^5^ The authors performed lineage-labeling studies of adult satellite cells in the mouse and show that the lineage reporter protein is packaged into EVs purified from satellite cell-derived myoblast cultures. These data confirm previous work published by this group.^6^ They next asked whether the EV packaged reporter is taken up by myofibers during load-induced hypertrophy. To distinguish between EV contribution versus satellite cell fusion with the myofiber syncytium, ability of cell fusion was compromised in satellite cells via genetic inactivation of Neural Wiskott-Aldrich Syndrom Protein (N-WASP), a fusion essential protein. Compared to controls, no increase in myonuclei was detected during the first week of load-induced hypertrophy in single myofibers with N-WASP^-^ satellite cells. Yet, the lineage label of the fusion-compromised satellite cells was detected in the myofiber syncytium. Using a cell culture approach, the authors next asked whether satellite cell-derived EVs can deliver cargo to myotubes. EVs were purified from lineage-labeled satellite cell-derived myoblast cultures and incubated with myotubes. Subsequently, the lineage-label was detected in the myotubes, leading the authors to conclude that satellite cells can communicate with myofibers during load-induced hypertrophy via EVs. This EV-mediated cargo delivery to myofibers parallels the authors’ previous findings on satellite cell-derived EV communication to fibroblasts.^6^

Next, the authors sought to identify genes in muscle tissue that are potentially modulated by satellite cell EV cargo during load-induced hypertrophy. Transcriptional profiling of overloaded muscle tissue in the presence or absence of satellite cells (via genetic cell ablation) revealed that the extracellular matrix regulator matrix metalloproteinase (*Mmp*)-9 is differentially expressed. Theorizing that satellite cells may deliver microRNAs (miRNAs) targeting *Mmp9* to myofibers via EVs, the authors turned to miRNA profiling of EVs purified from satellite cell-derived myoblasts. The authors discovered several miRNAs with either confirmed 3′ UTR target in *Mmp9* or experimentally confirmed *Mmp9* targeting. These findings are consistent with (and extend the authors previous finding of) satellite cell EV cargo containing miRNA-206, which also targets *Mmp9.*^6^ Cell culture experiments provide further evidence that incubating myotube-stage cultures with myoblast-derived EVs downregulated *Mmp9* expression. These data combined with the observations of transcriptomic changes in satellite cell-depleted, overloaded muscle tissue, have led the authors to propose that EVs originating from satellite cells can modulate the transcriptional program of myofibers.

This novel report that satellite cells may exert fusion-independent functions beyond their well-characterized fusion-dependent roles in load-induced hypertrophy merits further validation. To confirm fusion-independent roles of satellite cells in load-induced muscle hypertrophy, it will be necessary to revisit these studies utilizing an alternative genetic model to fully abolish satellite cell fusion. As demonstrated by the authors, fusion is delayed in N-WASP^-^ satellite cell-derived myoblast cultures but not eliminated.^5^ Indeed, the authors performed long-term load-induced hypertrophy studies and show that N-WASP inactivation in satellite cells does not compromise gains in muscle mass, nor myonuclear accretion. Hence, further examination of fusion-dependency of cargo delivery from satellite cells to myofibers is warranted. For example, genetic inactivation of the muscle-specific gene myomaker resulted in a complete block on myoblast fusion.^7^ Therefore, lineage-tracing of myomaker satellite cells in load-induced hypertrophy may establish supportive or alternative data for the reported remarkable fusion-independent roles of satellite cells. Furthermore, a direct approach of engineering satellite cells blocked from EV production and/or release would not just help advance investigating satellite cell-derived EVs in muscle biology but present a tremendous boost for EV research at large. This approach is currently hindered by technical challenges imposed by the complex biology of vesicle trafficking. An alternative approach may be to functionally test satellite cell-derived EV cargo in the regulation of the myofiber. One would predict that satellite cell-specific elimination of miRNAs targeting *Mmp9* alters extra-cellular matrix remodeling in overloaded myofibers.

While fundamental research investigating whether satellite cell-derived EVs and their cargo are necessary modulators of load-induced myofiber hypertrophy is much needed, such a signaling platform could potentially constitute a novel, exciting regulatory axis for muscle tissue even beyond load-induced hypertrophy. A remarkable, yet to date unexplained phenomenon occurs in many skeletal muscle exercise regimens where eccentric contractions will induce substantial increases in satellite cells.^8^ In the absence of matching satellite cell fusion with myofibers, could these cell increases facilitate fusion-independent roles via EV-mediated signaling to myofibers? Moreover, are satellite cell-derived EVs contributing to skeletal muscle regulation during homeostasis, and in aging and disease? Lineage-tracing studies in sedentary adult mice have suggested pervasive fusion of satellite cells for homeostatic maintenance of myofibers.^9^^,^^10^ Fusion-independent communication via EVs during homeostasis could provide an alternative explanation for these observations. In light of these broad, challenging implications, future functional studies on satellite cell-derived EVs are of great interest and warranted.

## Conflict of Interest Statement

None declared.
